# New challenges for polio eradication in Russia

**Published:** 2011-01-10

**Authors:** NI Romanenkova, MA Bichurina, NR Rozaeva, OE Ivanova, TP Eremeeva, ML Yakovenko

**Affiliations:** 1 Department of Virology, Institut Pasteur of Saint-Petersburg, Saint-Petersburg, Russia; 2 M.P.Chumakov Institute of Poliomyelitis and Viral Encephalitides, Moscow, Russia

## 

During the post-eradication era of Global Polio Eradication, the role of supplementary virological surveillance of groups at risk for the disease increases. The goal of all types of surveillance is to detect possible wild poliovirus importation and evaluate vaccine-derived polioviruses circulation. The large outbreak of poliomyelitis caused by the wild poliovirus of type 1 in Tadjikistan and registration of some cases of poliomyelitis among Tadjik children who have recently arrived in Russia requires the systematic virological surveillance of the children from Tadjik migrants’ families.

In 2006-2009 the Sub-national Polio Laboratory investigated the stool samples from 272 children from migrants’ families. The strains of polioviruses of different serotypes were isolated from 10 children including 5 children from Tadjikistan. All strains were classified as vaccine-like strains. The percentage of isolation of polioviruses was higher among the healthy children from migrants’ families (3.7%) than among the patients with acute flaccid paralysis (AFP) and healthy children who had contacts with them (1.9%). More prolonged excretion of polioviruses after vaccination was also demonstrated by the children from migrants’ families.

In 2010 the laboratory examined the stool samples from 112 children, including 86 children from Tadjik migrants’ families. Nine polioviruses were isolated from 86 Tadjik children (10.5%). Three strains of polioviruses of type 1 isolated from three healthy Tadjik children were classified as non-Sabin-like according to the results of ELISA-test and PCR with specific primers. The genomic sequencing confirmed that the isolated viruses were wild polioviruses of type 1. The supplementary immunization targeting to avoid the wild poliovirus transmission was held at a nursery school.

The systematic virological surveillance of AFP patients and children from the groups at risk for the disease and adequate epidemiological measures are indispensable in order to prevent wild poliovirus transmission and indigenous circulation of wild polioviruses after its importation to polio free countries.

